# Generative Model of Brain Microbleeds for MRI Detection of Vascular Marker of Neurodegenerative Diseases

**DOI:** 10.3389/fnins.2021.778767

**Published:** 2021-12-16

**Authors:** Saba Momeni, Amir Fazlollahi, Leo Lebrat, Paul Yates, Christopher Rowe, Yongsheng Gao, Alan Wee-Chung Liew, Olivier Salvado

**Affiliations:** ^1^Commonwealth Scientific and Industrial Research Organisation (CSIRO) Data61, Brisbane, QLD, Australia; ^2^School of Engineering and Built Environment, Griffith University, Nathan, QLD, Australia; ^3^Commonwealth Scientific and Industrial Research Organisation (CSIRO) Health and Biosecurity, Australian E-Health Research Centre, Brisbane, QLD, Australia; ^4^Queensland Brain Institute, The University of Queensland, Brisbane, QLD, Australia; ^5^Department of Geriatric Medicine, Austin Health, Heidelberg, VIC, Australia; ^6^Department of Molecular Imaging and Therapy, Austin Health, Heidelberg, VIC, Australia; ^7^The Florey Department of Neuroscience and Mental Health, The University of Melbourne, Parkville, VIC, Australia; ^8^School of Information and Communication Technology, Griffith University, Nathan, QLD, Australia; ^9^Department of Nuclear Medicine, Centre for PET, Austin Health, Heidelberg, VIC, Australia

**Keywords:** generative adversarial network, cerebral microbleed, data augmentation, deep learning, SWI images, synthetic data

## Abstract

Cerebral microbleeds (CMB) are increasingly present with aging and can reveal vascular pathologies associated with neurodegeneration. Deep learning-based classifiers can detect and quantify CMB from MRI, such as susceptibility imaging, but are challenging to train because of the limited availability of ground truth and many confounding imaging features, such as vessels or infarcts. In this study, we present a novel generative adversarial network (GAN) that has been trained to generate three-dimensional lesions, conditioned by volume and location. This allows one to investigate CMB characteristics and create large training datasets for deep learning-based detectors. We demonstrate the benefit of this approach by achieving state-of-the-art CMB detection of real CMB using a convolutional neural network classifier trained on synthetic CMB. Moreover, we showed that our proposed 3D lesion GAN model can be applied on unseen dataset, with different MRI parameters and diseases, to generate synthetic lesions with high diversity and without needing laboriously marked ground truth.

## Introduction

Cerebral microbleeds (CMB) are small hypointense spots on brain MRI susceptibility-weighted imaging (SWI), known as chronic blood products in normal (or near-normal) brain tissues (Greenberg et al., 2009). Since CMB are valuable biomarkers to explain cognitive impairment and diagnose vascular diseases, automated CMB detection methods have seen a recent increase in interest. Most automated CMB detections use machine learning (Barnes et al., 2011; Bian et al., 2013; Fazlollahi et al., 2015; Roy et al., 2015), including deep learning methods, achieving superior performance by increasing the sensitivity to 95.8 and reducing the number of false positives to 1.6 (Dou et al., 2016; Zhang et al., 2017; Liu et al., 2019; Faryna et al., 2021). However, they are seldom used for clinical application because of the high number of false positives and the limited evidence that they generalize to different acquisition protocols (e.g., different SWI parameters, scanners, or cohorts).

Common challenges for detecting CMB with machine learning include the limited availability of ground truth, their relatively low prevalence, small size, variations in shape, intensity, and size, and the high number of mimics, such as vessel cross-sections that result in many false positive (FP) detections. To compensate for the limited ground truth, data augmentation methods almost always include flipping, rotation, and sometimes noise addition or gamma correction (Dou et al., 2016; Liu et al., 2019). Random majority sample (negative class) reduction and cost-sensitive learning are two other methods used to address the issue of imbalanced data when typically only a few CMB are present in a MRI scan and only a fraction of subjects have CMB (Wang et al., 2017; Zhang et al., 2017).

Generating synthetic data as a data augmentation strategy has several advantages. The size of the training data can be made as large as desired, as long as negative cases exist where synthetic positives could be added. The variety of the synthetic data could be arbitrarily increased to cover a larger training space than that of real cases. Finally, synthetic data generation requires neither domain expertise nor enrolling actual subjects, saving cost, and avoiding any ethical issues.

Generative adversarial networks (GANs) (Goodfellow et al., 2014) is a technique for generating fake data with a distribution similar to that of real data. GAN comprises two neural networks competing against each other: a discriminator and a generator. The generator creates fake data and maximizes the confusion of the discriminator to distinguish real from fake data, which the discriminator tries to identify (Kazeminia et al., 2020).

Several GAN models have been applied to medical applications (Frid-Adar et al., 2018; Iqbal and Ali, 2018; Zhao et al., 2018; Wu et al., 2020). Frid-Adar et al. (2018) proposed three deep convolutional GANs to generate three classes of liver lesions (cysts, metastases, and hemangiomas). The generated samples were found to be beneficial for classifying lesions on computed tomography (CT). Zhao et al. (2018) proposed a forward and backward GAN (F&BGAN) to improve lung nodule classification, with the aim of enhancing the synthetic data quality. Wu et al. (2020) proposed a U-Net-based generator architecture to generate or remove lesions on high-resolution mammography images, which could leverage the background information. They showed that, for malignancy classification, by adding the synthetics to the real data, the area under the receiver operating characteristic (ROC) curve increased from 0.829 to 0.846.

Cross-modality synthesis methods have been proposed in several works (Bi et al., 2017; Wolterink et al., 2017; Hiasa et al., 2018). Most have deployed deep learning-based methods to learn end-to-end non-linear mapping from magnetic resonance images to CT images or positron emission tomography images. CycleGAN has shown capability to synthesize unpaired dataset between different modalities (Zhu et al., 2020).

Conditional GAN (CGAN) was proposed by Mirza and Osindero (2014) and Jin et al. (2018), adding a condition as an input to both the generator and the discriminator. Close to our work, Jin et al. (2018) applied a three-dimensional (3D) CGAN to generate synthetic nodules on CT images that improved the performance of a deep learning model for pathological lung segmentation. They employed volume as a condition but added fake lesions only to locations which had real lesions removed, with a heuristic technique to blend the new fake lesions to the modified scans. Removing a lesion could create artifacts and preclude generating data in healthy areas, two limitations that we address with our simpler method, where a lesion mask that can be multiplied at any location to add fake lesions is generated.

In Momeni et al. (2018, 2021), we proposed an analytical model to create synthetic microbleeds for SWI MRI images. They hypothesized that CMB are Gaussian-liked structures spread all over the brain. 3D Gaussian lesions were created, in high resolution, with randomized shapes to simulate variation in shape and volume. The partial volume effect was simulated by down-sampling the patch that was multiplied at random locations where there was no actual lesion. Their synthetic dataset was compared to traditional data augmentation and synthetic minority oversampling technique (SMOTE) (Chawla et al., 2002). The results showed that synthetic CMB (sCMB) improved CMB classification with less than nine FP per scan using a random forest classifier. We are now improving on that work by learning the lesion shape and appearance, which can be adapted to the background using GAN.

A preliminary work using CycleGAN data augmentation model to generate CMB was recently reported by Faryna et al. (2021). The authors used a series of complex healthy pathological transformations conditioned by a mask where fake lesions should be created. One drawback of that approach is the need to delineate lesions (our method requires only point locations), and the processing of the whole dataset might also affect otherwise healthy locations. CycleGAN (Zhu et al., 2020) was also applied to detect CMB associated with traumatic brain injury. During the training, besides adversarial and CycleGAN loss, the authors considered an abnormality mask loss to preserve brain structure outside of pathological regions. Adding synthetic data improved the performance, but an abnormality mask was required.

In this paper, we propose a novel 3D LesionGAN method that uses the background and lesion volume as conditions to generate synthetic CMB using GAN. A mask is generated, which can be multiplied at any location within the brain on any unseen MRI dataset. We trained a convolutional neural networks (CNN) classifier for CMB classification from whole SWI images. We used sCMB during training and real lesions for testing, including a different dataset. The main contribution of this paper is twofold: (1) we investigated whether a new GAN model could create synthetic CMB on SWI conditioned on location and volume and (2) we investigated whether synthetic lesions generalize to a new unseen dataset with a different population, MRI parameters, and pathologies.

## Materials and Methods

### Generative Adversarial Network

GAN have been introduced as a novel way to generate synthetic data by Goodfellow et al. (2014). They consist of two competing parts, a generator is trained to fool a discriminator by generating fake images, while the discriminator is trained to distinguish between real and fake images. More formally, the generator learns to transform a prior noise distribution *p*_*z*_(*z*) to the data distribution *p*_*g*_(*x*). The discriminator classifies whether a data sample has been generated from the generator (*p*_*g*_(*x*)) or from the real dataset *p(x)*. The generator parameters are optimized to minimize log(1−*D*(*G*(*z*))), whereas those of the discriminator are to maximize *D*(*x*) and 1−*D*(*G*(*z*)), following the two-player min–max game with value function *V*(*G*, *D*):


(1) $$\min_{G}\max_{D}V\left(G,D\right)=\min_{G}\max_{D}E_{x∼p_{d\,a\,t\,a}}[\left[\text{log}\left(D\left(x\right)\right]\right]+E_{z∼p_{z}}[\left[\text{log}\left(1-D\left(G\left(z\right)\right)\right]\right]$$

where *D*(*x*) represents the probability from the discriminator that *x* belongs to the real data and *D*(*G*(*z*)) is the probability that the discriminator classifies the generated synthetic data *G*(*z*) as real. *G* and *z* refer to the generator and input noise, respectively.

### Synthetic Microbleed Generation Using LesionGAN

**FIGURE 1:**
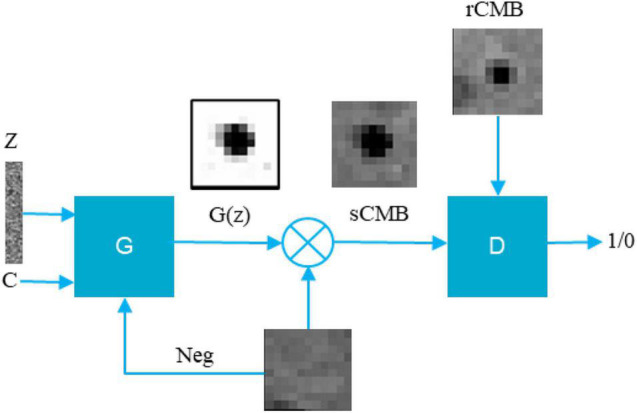
Proposed conditional LesionGAN pipeline to generate sCMB. Z, noise; C, condition; G(z), cerebral microbleed partial volume mask; Neg, negative patch; G, generator; D, discriminator.

We hypothesized that the shape and the appearance of a CMB depend on the surrounding tissues. We thus add as an input to the generator a negative patch (a patch with no CMB randomly sampled from the MRI datasets), where the synthetic CMB will be added. We also include as an input to the generator the desired CMB volume. The output of the generator is partial volume mask *G*(*z*) of size 11 × 11 × 11, where the lesion is centered with intensity between 0 and 1, whereas the background around the lesion is equal to 1. The CMB mask *G*(*z*) can then multiply the negative patch (provided to the generator as an input), thereby creating a patch with a synthetic CMB (sCMB). The discriminator is trained to classify whether its input is coming from the generator (sCMB) or from a CMB patch of the same size containing a real CMB (rCMB). Figure 1 describes this pipeline.

The generator is trained to generate a lesion whose volume matches the input volume by adding a new loss:


(2) $$L\,o\,s\,s_{v\,o\,l\,u\,m\,e}=\frac{1}{N}\,\sum_{i=1}^{N}\left|\frac{V_{f\,a\,k\,e}^{i}-V_{i\,n}^{i}}{V_{i\,n}^{i}}\right|$$

where $V_{\text{fake}}^{i}$ and $V_{\text{in}}^{i}$ are the *i*^*th*^ fake and *i*^*th*^ input volume, respectively. $V_{\text{fake}}^{i}$ is computed by summing up 1-*G*(*z*) for each generated synthetic CMB mask.

To enforce the sCMB mask to blend with the background when multiplying by *G*(*z*), we force all the edge voxels of the mask *G*(*z*) to be equal to 1 by adding a border loss:


(3) $$L\,o\,s\,s_{b\,o\,r\,d\,e\,r}=\frac{1}{K}\,\sum_{\left(i,j,k\right)\in B}\left|1-G\,{\left(z\right)}_{i\,j\,k}\right|$$

where *B* is a set of the voxels on the border of *G*(*z*), and *K* is the number of samples in *B*. Eventually, the total losses for the generator and discriminator are:


(4) $$L\,o\,s\,s\,G=\min_{G}\left(E_{z∼p_{z}\,\left(z\right)}\,\left[\left[\text{log}\,\left(1-D\,\left(G\,\left(z,c\right)\right)\right)\right]\right]\right)+L\,o\,s\,s_{b\,o\,r\,d\,e\,r}+L\,o\,s\,s_{v\,o\,l\,u\,m\,e}$$

(5) $$LossD=\max_{D}(E_{x∼p_{d\,a\,t\,a}\,\left(x\right)}\left[\left[\text{log}\left(D\left(x\right)\right)\right]\right]+E_{z∼p_{z}\,\left(z\right)}\left[\left[\text{log}\left(1-D\left(G\left(z,c\right)\right)\right)\right]\right])$$

**FIGURE 2:**
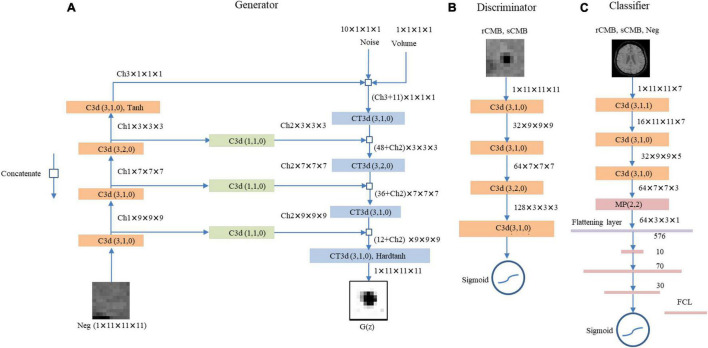
The proposed LesionGAN generator, discriminator, and cerebral microbleed (CMB) classifier are shown. **(A)** Generator: G(z), generated CMB mask; Neg, negative patch; CT3d (k,s,p), transposed convolution with kernel size, stride, and padding of k, s, p; C3d (k,s,p), convolutional layer with kernel size, stride, and padding of k, s, p. Ch1, Ch2, and Ch3 are channel numbers set to {6,1,8}. ReLU is defined as an activation function for all layers. **(B)** The discriminator with LeakyReLU and sigmoid as activation functions for hidden and last layers. The input patches for discriminator are augmented rCMB. **(C)** The CMB classifier with ReLU and sigmoid function as applied activation functions for the hidden and last layers, respectively. FCL, fully connected layer; MP (k,s), max pooling with kernel size and stride of k and s, respectively.

The final generator and discriminator networks are shown in Figures 2A,B.

### Cerebral Microbleeds Classifier

We trained a classifier to detect CMB from SWI images (Figure 2C). It comprises three CNN layers (kernel size of 3) with 16, 32, and 64 feature maps, respectively, and three fully connected layers with 10, 70, and 30 neurons (empirically chosen after testing many combinations during validation on a separate set of synthetic data). We used max pooling with size 2, batch normalization, and 50% dropout. Data term was binary cross-entropy optimized with Adam (Kingma and Ba, 2017). Activation functions included rectified linear unit (ReLU) and sigmoid (Ramachandran et al., 2017) as shown in Figure 2C. The CMB classification performance is reported using 10-fold cross-validations (on the subjects) with an ensemble average of 10 networks.

### Dataset

Two datasets were used. The first one (DS1) is from the Australian Imaging Biomarkers and Lifestyle (AIBL) dataset as described in Momeni et al. (2021), and the second one (DS2) is from the MICCAI Valdo challenge in 2021. The data comprising DS1 are freely available online at https://doi.org/10.25919/aegy-ny12.

#### Australian Imaging Biomarkers and Lifestyle Dataset (DS1)

Approval for the study was obtained from the Austin Health Human Research Ethics Committee and St. Vincent’s Health Research Ethics Committee, and written informed consent was obtained. All subjects underwent an anatomical T1-weighted (T1w) and a SWI acquisition on a 3-T Siemens TRIO scanner, where SWI was reconstructed online using the scanner system (software VB17). More details about the dataset can be found in previous publications (Momeni et al., 2021). We considered definite CMB for the true positive class. Table 1 shows a summary of the data used.

**TABLE 1:** Gathered information from DS1.

Data type	Scan number AD/MCI/HC/unknown CMB number AD/MCI/HC/unknown	Subject number/scan number/CMB number	Age average (F/M) ± standard deviation
Whole dataset	44/38/151/4 46/43/144/2	141/263/235	74 ± 7/74 ± 4
At least one definite CMB	13/17/43/2 33/35/105/2	41/75/175	77 ± 8/76 ± 6
No CMB	22/15/81/2	70/146/0	73 ± 6/74 ± 8
Just possible CMB	9/6/27/0 13/8/39/0	30/42/60	74 ± 5/73 ± 3

*F, female; M, male; AD, Alzheimer’s disease clinical diagnosis; MCI, mild cognitive impairment; HC, healthy control.*

#### MICCAI Valdo Challenge Dataset (DS2)

We used the MICCAI Valdo Challenge in 2021^1^ to investigate whether the synthetic lesion learnt from DS1 could generalize to another dataset with different MRI parameters, subject population, and pathologies. DS2 includes 72 scans with a different resolution than that of DS1: 0.44 × 0.44 × 4 mm^3^. Out of 72 scans, 50 have 235 marked CMB, while the remaining 22 have no CMB. We excluded one scan which had an unusual number of lesions (72) compared to the rest of the patients (the second and the third highest number of lesions were 26 and 20; most have a few).

#### Distribution of Lesion Volume

To compute the volume of real lesions from DS1 and DS2, for each rCMB patch (7 × 7 × 7), a mixture of distributions comprising a Gaussian to model brain tissues and a uniform distribution for modeling the outliers (blood vessels and CMB) were fitted to the intensity histogram: we defined the intensity of the patch as *G*(,σ) + *U*(0,255), with *G* and *U* being the normal and uniform distribution, respectively, and optimized using the expectation maximization method for μ and σ. A maximum posterior classification created a mask of the lesion from which the fraction of the CMB could be computed by using a standard partial volume model and summation over all the pixel to obtain the volume for the patch.

**FIGURE 3:**
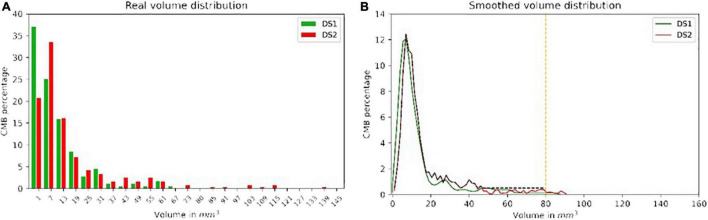
The real and smoothed volume distribution from both DS1 **(A)** and DS2 **(B)** with green and red color, respectively. The black dash line shows the distribution ultimately used to generate the sCMB from DS2.

The real CMB volume distributions were similar in both datasets. However, DS2 included lesions with larger volumes than those in DS1, with a larger principal mode (1 mm^3^ for DS1 vs. 7 mm^3^ for DS2). When generating sCMB to train the classifier, the distribution of volume was smoothed and limited to 80 mm^3^ for consistency between the two datasets. For DS2, a minimum volume probability of 0.5% was used to guarantee that training samples included all possible volumes between 0 and 80 mm^3^. Figure 3 shows the real and smoothed volume distribution for both DS1 and DS2.

### Preprocessing

For DS1 and DS2, standard bias field correction and histogram matching were applied (Momeni et al., 2021). Histogram matching and resampling were done by using MRtrix (mrhistmatch 3.0) and Mirror package^2^ with the same reference image used for DS1 histogram matching. For DS2, the resolution was interpolated to match DS1: 0.93 mm × 0.93 mm × 1.75 mm, resulting in a final volume of 176 × 256 × 80 voxels.

## Results and Experiments

We investigated whether using the proposed sCMB for training could improve the performance of the classifier for detecting real lesions compared to other recent data augmentation approaches. We then investigated whether the generator trained with real lesions from DS1 could be used to train a classifier to detect lesions from DS2 without the need for any ground truth from DS2.

### Training LesionGAN

**FIGURE 4:**
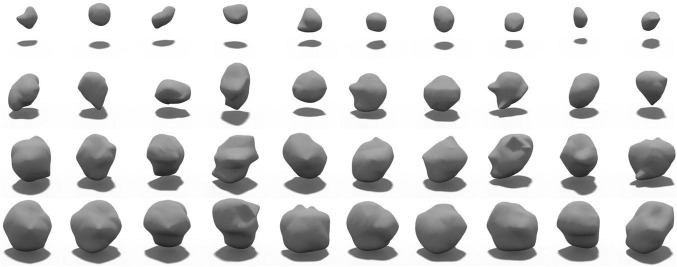
Samples of generated sCMB from our proposed LesionGAN model for different volumes. Each row shows different sCMB with 10 different noises for a specific volume such as 5, 15, 20, and 30 mm^3^, respectively.

In this experiment, 50% of subjects with possible CMB and 30% of subjects with no rCMB were used to train LesionGAN. Real patches were augmented using rotation (90, 180, and 270°) and flipping around the x, y, and z axes, resulting in approximately 5,000 patches. Negative patches (Figure 1) were selected randomly in locations with no real CMB present, respecting the same distribution within and distance from brain tissues as observed with real lesions (Momeni et al., 2021). All inputs (negative patches and volume) were normalized between 0 and 1. For the generator, latent variables were randomly generated from a normal-centered distribution with unit variance. The Adam optimizer (Kingma and Ba, 2017) was used with parameters β1 = 0.5 and β2 = 0.999. A learning rate of 0.0002 with 2,500 epochs and a batch size of 64 was used for both generator and discriminator training. Figure 4 shows examples of the generated sCMB for four different volumes and 10 different random noise.

**FIGURE 5:**
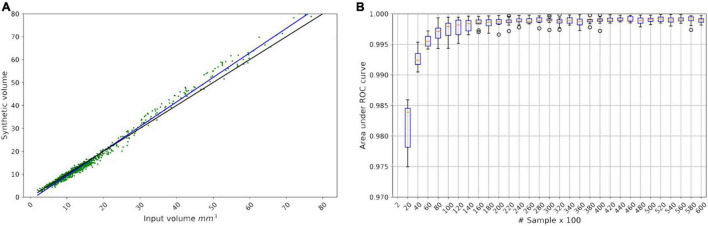
**(A)** Linear regression model for the LesionGAN by deploying volume value as a condition. The black line is identity line; the blue line is the fitted linear model (*y* = 1.07*x* - 1.28) between real and generated volumes. The green scatter points show the generated synthetic volume for each input volume. **(B)** Area under the receiver operating characteristic curve from saturating the classifier by applying 60,000 samples in 30 steps, each step adding 2,000 samples.

To validate the volume condition, we generated 1,000 fake CMB mask (*G*(*z*)) from the smoothed volume distribution shown in Figure 3 and regressed the resulting *G*(*z*) volume with the target volume provided as input to the generator. The result is shown in Figure 5A with R-square of 97.99%.

Because we could generate as many lesions as desired, we investigated the number of samples required to saturate the performance of the classifier. Figure 5B shows that the performance of the classifier did not improve beyond 20,000 samples (10,000 positives and 10,000 negatives) with an area under the curve (AUC) of 0.9983. All experiments used 10-fold cross-validation and an ensemble of 10 classifier networks.

### Comparison of Data Augmentation Models for Patch Classification

We classified patches with no CMB (Neg) and real CMB using various data augmentation methods. In this part, all experiments were done by using DS1 comprising 50% of the subjects with possible CMB, 70% of the subjects with no lesion, and all the subjects with definite rCMB.

**FIGURE 6:**
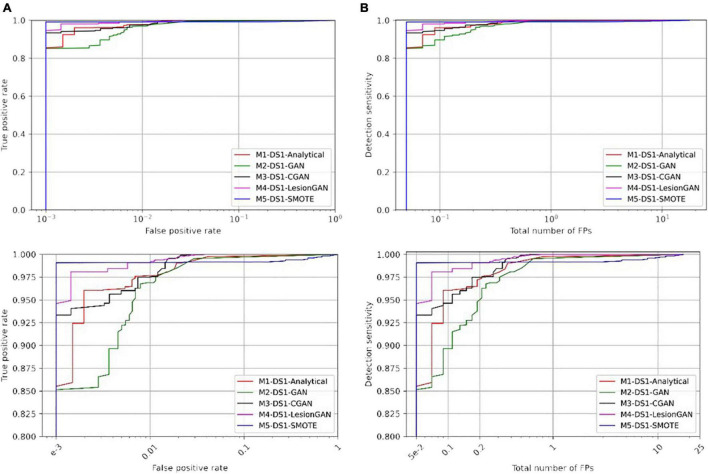
Comparing receiver operating characteristic (ROC) **(A)** and free-response ROC **(B)** curves of five different training models for cerebral microbleed classification on the patch after training on 20,000 samples. The bottom panel shows the zoomed version.

We compared five data augmentation models: model 1: the synthetic analytical model described by Momeni et al. (2021) (M1-DS1-Analytical), model 2: GAN without any condition (M2-DS1-GAN), model 3: GAN with the lesion volume as a condition (M3-DS1-CGAN), model 4: LesionGAN that includes the background and volume as inputs to the generator (M4-DS1-LesionGAN), and model 5: synthetic minority oversampling technique (M5-DS1-SMOTE). For all models, data augmentation resulted in 10,000 positive and 10,000 negative patches. The results are from 10-fold cross-validations (on the subjects) repeated five times with different data order and an ensemble of 10 networks. Training was done for 200 epochs with a learning rate of 10^–5^ and a batch size of 128. The optimizer was Adam with the same parameters as mentioned above. Figure 6 shows the ROC and free-response receiver operating characteristic (FROC) curves. Table 2 shows the specificity and number of FP for 95% sensitivity.

**TABLE 2:** Results of cerebral microbleed patch classification.

Training model	AUC	Sensitivity (95%)	
		Spe	Number of FP
M1-DS1-Analytical	0.9992 ± 7*e*−4	0.9977 ± 1*e*−3	0.091 ± 5*e*−2
M2-DS1-GAN	0.9989 ± 4*e*−4	0.9933 ± 3*e*−3	0.283 ± 8*e*−2
M3-DS1-CGAN	0.9995 ± 2*e*−4	0.9965 ± 4*e*−3	0.109 ± 8*e*−2
M4-DS1-LesionGAN	**0.9996** ± **2e−4**	0.9989 ± 8*e*−4	0.069 ± 4*e*−2
M5-DS1-SMOTE	0.9946 ± 2*e*−3	**0.9990** ± **1e−10**	**0.051** ± **2e−12**

*Shown are the average ± standard deviation of five draws using DS1. AUC, area under the receiver operating characteristic curve; Spe, specificity; FP, average number of false positives per scan. Bold represents the best score for each criteria.*

In Table 2, the highest AUC was found for M4-DS1-LesionGAN (AUC = 0.9996), followed by M3-DS1-CGAN (AUC = 0.9995), M1-DS1-Analytical (AUC = 0.9992), and M2-DS1-GAN (AUC = 0.9989), while the M5-DS1-SMOTE (AUC = 0.9946) obtained the lowest AUC, whose specificity dropped quickly above 98% sensitivity. Comparing the specificity and number of FP for 95% sensitivity, the order of the performance was as follows: M5-DS1-SMOTE, M4-DS1-LesionGAN, M1-DS1-Analytical, M3-DS1-CGAN, and M2-DS1-GAN. The two best models with similar results were M5-DS1-SMOTE and M4-DS1-LesionGAN, and the latter had the highest specificity score of ∼0.9990 and the lowest number of FP ∼0.051.

### Whole Susceptibility-Weighted Imaging Cerebral Microbleeds Classification

**FIGURE 7:**
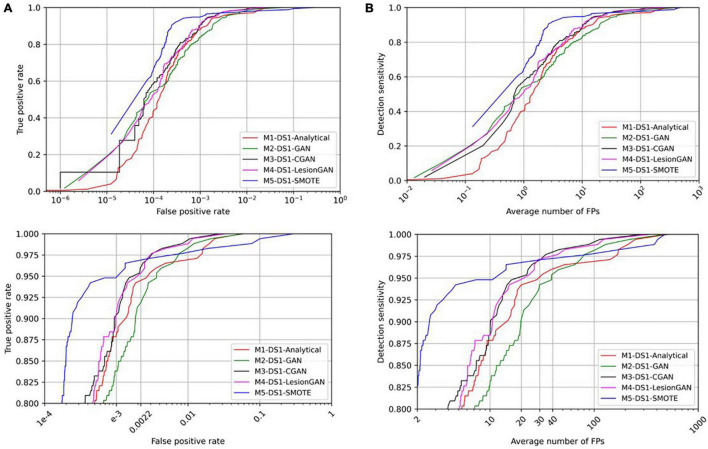
Comparing receiver operating characteristic (ROC) **(A)** and free-response ROC **(B)** curves for cerebral microbleed classification on the whole SWI by 20,000 training samples. The bottom panel shows the zoomed version.

To evaluate the clinical application of lesion detection, CMB detection was performed on whole MRI. Radial symmetry transform (RST) (Loy and Zelinsky, 2003) was applied as a first screening step to reduce the number of lesion candidates, using a radius range of 1–4 and a radial strictness pixel of 2. The RST filtering used a Gaussian kernel with standard deviation of 0.8 pixel. RST only missed one lesion overall (sensitivity of 99.43%) and identified approximately 7,000 candidate locations for each scan, down from about 750,000 possible locations per scan. The results after applying a classifier trained using the different data augmentation models are shown in Figure 7 with ROC and FROC curves. Quantitative performances are summarized in Table 3 for 95% sensitivity.

**TABLE 3:** Results of cerebral microbleed (CMB) classification on the whole SWI training on 20,000 samples and test on the DS1.

Training model	AUC	Sensitivity (95%)		
		Spe	Number of false positive/scan	Number of DPCMB
M1-DS1-Analytical	0.9990	0.9973	30	110
M2-DS1-GAN	0.9987	0.9964	40	108
M3-DS1-CGAN	**0.9996**	0.9982	20	111
M4-DS1-LesionGAN	**0.9996**	0.9980	22	**118**
M5-DS1-SMOTE	0.9918	**0.9989**	**11**	77

*AUC, area under the receiver operating characteristic curve; Spe, specificity; DPCMB, detected possible CMB. Bold represents the best score for each criteria.*

In Table 3, the highest AUC was obtained for both M3-DS1-CGAN and M4-DS1-LesionGAN (AUC = 0.9996) followed by M1-DS1-Analytical (AUC = 0.9990) and M2-DS1-GAN (AUC = 0.9987), while M5-DS1-SMOTE had the lowest performance (AUC = 0.9918).

With respect to specificity and number of FP for 95% sensitivity, the ranking of the models based on the performance was as follows: M5-DS1-SMOTE, M4-DS1-LesionGAN, M3-DS1-CGAN, M1-DS1-Analytical, and M2-DS1-GAN. All models had a specificity above 0.99. M5-DS1-SMOTE obtained the highest specificity score of ∼0.9989 and the lowest FP number of 11. M4-DS1-LesionGAN and M3-DS1-CGAN reported similar results with a specificity of 0.9980 and 0.9983 and FP of 22 and 20, respectively.

We computed the number of possible CMB that were detected by each method for 95% sensitivity. The best method was M4-DS1-LesionGAN with 118, followed by M3-DS1-CGAN (111), M1-DS1-Analytical (110), and M2-DS1-GAN (108), while M5-DS1-SMOTE detected the fewest (77).

### Comparison With Other Works

In Table 4, we compare the performance of our classifier with four other recent approaches using deep learning network (DLN). Dou et al. (2016) used 3D convolutional neural network after applying rotation, flipping, and translation to augment the training data. Liu et al. (2019) applied 3D RST as a screening method and used a convolutional neural network to detect CMB. SWI and QSM, including the phase, were used. They reported 95.8% sensitivity, 1.6 FP, and 70.9% precision. Zhang et al. (2017) used a sliding neighborhood and random undersampling with a seven-layer DLN (including four sparse autoencoder layers) and reported 95.3% sensitivity with 93.3% specificity. We estimated 492 FP from other results. For a fair comparison, we estimated the results for the number of FP, specificity, and precision using the same sensitivity reported by each of the corresponding papers (Table 4).

**TABLE 4:** State-of-the-art performance for cerebral microbleed (CMB) classification.

Reported results							Our results using (M4-DS1-LesionGAN) for the same Sen as published		
	
Method	MRI	Number of subjects/ number of CMB	Sen %	Spe %	Pre %	Number of FP per scan	Spe %	Pre %	Number of FP per scan
Liu et al. (2019)	Complex SWI, QSM	220/1641	95.8	N/A	**70.9**	**1.6**	99.79	0.798	25.87
Dou et al. (2016)	SWI/3T	20/NA	93.1	N/A	**44.1**	**2.7**	99.88	14.02	13.37
Zhang et al. (2017)	SWI/3T	10/NA	95.3	93.3	N/A	492^a^	**99.78**	0.867	**23.24**
Faryna et al. (2021)	SWI	60/NA	90	N/A	N/A	31.4, 27.4	99.90	16.47	**9.82**

*Sen, sensitivity; Spe, specificity; Pre, precision; FP, false positive per scan.*

*^a^Inferred from other reported results. Bold represents the best score for each criteria.*

Our proposed LesionGAN method had more FP than the original published works (Dou et al., 2016; Liu et al., 2019): 25.87 and 13.37 compared to 1.6 and 2.7, respectively, but based only on SWI magnitude compared to multi-channel processing used by the two references. Faryna et al. (2021) applied CMB classification on two different training datasets. The first dataset included reals, augmented reals, and synthetics, and the second was without augmented reals. For the sensitivity of 90%, they reported 27.4 and 31.4 FP for the training dataset with and without augmented reals, respectively. For the same sensitivity (90%), our model resulted in a much higher specificity of 99.90% and fewer FP: 9.82. Compared to that of Zhang et al. (2017), our model had fewer FP and higher specificity with 23.24 and 99.78% compared to the 492 and 93.3%, respectively. Our reported precision numbers are in the low range of values, which means that the number of FP from our method is high for each tested scan. By using more complex classifier and multi-channel dataset as applied in Liu et al. (2019), it should be possible to reduce the FP and increase the precision.

### Applying LesionGAN on Unseen MRI Dataset

We tested our classifier to detect CMB on a new unseen dataset (DS2). This is the most challenging case: detecting lesions using a model trained from a completely different dataset than the one used for training, in terms of scanner, MRI sequence, pathologies, image resolution, and patient population. A total of 20,000 samples were used for training in a balanced scheme, with the same classifier as described in section “CMB Classifier.”

First, we trained the classifier using DS1 and tested it on DS2. Model 6 corresponds to CGAN (conditioned on volume only: M6-DS1-CGAN), whereas model 7 corresponds to LesionGAN (volume and background as conditions: M7-DS1-LesionGAN). The classification results on the patch are shown in Table 5 (left panel).

**TABLE 5:** Results of cerebral microbleed (CMB) patch classification.

Training on the sCMB from DS1, test on the DS2				Training on the sCMB from DS2, test on the DS2			
**Training model**	**AUC**	**Sensitivity (95%)**		**Training model**	**AUC**	**Sensitivity (95%)**	
		**Spe**	**Number of FP**			**Spe**	**Number of FP**

M6-DS1-CGAN	0.9162	0.5574	10.68	M8-DS2-CGAN	0.9345	0.5034	11.98
M7-DS1-LesionGAN	0.9094	0.5298	11.37	M9-DS2-LesionGAN	**0.9416**	**0.6625**	**8.13**
M10-DS1-SMOTE	0.7881	0.2010	19.26				

*AUC, area under the receiver operating characteristic curve; Spe, specificity. Bold represents the best score for each criteria.*

**FIGURE 8:**
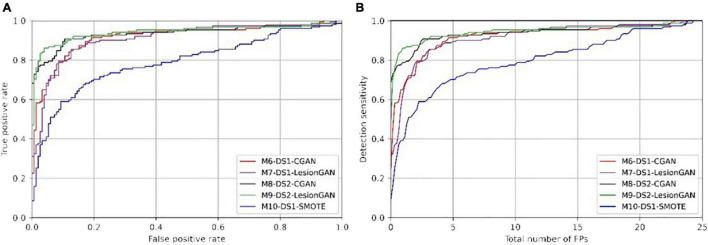
Receiver operating characteristic (ROC) **(A)** and free-response ROC **(B)** curves for cerebral microbleed classification on the patch for unseen MRI dataset (DS2).

We then used the GAN trained on DS1 and made the synthetic data (sCMB) using negative patches from DS2. Indeed, the classifier was thus trained using patches from DS2, but with synthetic lesions learnt from DS1. In other words, no ground truth from DS2 was used for training the classifier to detect lesions on DS2. The corresponding models are M8-DS2-CGAN and M9-DS2-LesionGAN. The performances are reported in Table 5 (right panel). Figure 8 shows the ROC and FROC curves for all the configurations.

In Table 5, the highest AUC was obtained with M9-DS2-LesionGAN (0.9416) and M8-DS2-CGAN (0.9345), followed by M7-DS1-CGAN (0.9162) and M6-DS1-LesionGAN (0.9094), revealing the effect of the different background and MRI appearance on the classifier performance. The lowest AUC was achieved by M10-DS1-SMOTE (0.7881), which performed poorly on the new dataset DS2.

Comparing the specificity and number of FP for 95% sensitivity, M9-DS2-LesionGAN had the best performance with 0.6625 specificity and 8.13 FP, followed by M6-DS1-CGAN (0.5574 specificity and 10.68 FP) and M7-DS1-LesionGAN (0.5298 specificity and 11.37 FP). Similarly, M10-DS1-SMOTE had the worst performance, by far, with 0.2010 specificity and 19.26 FP.

**FIGURE 9:**
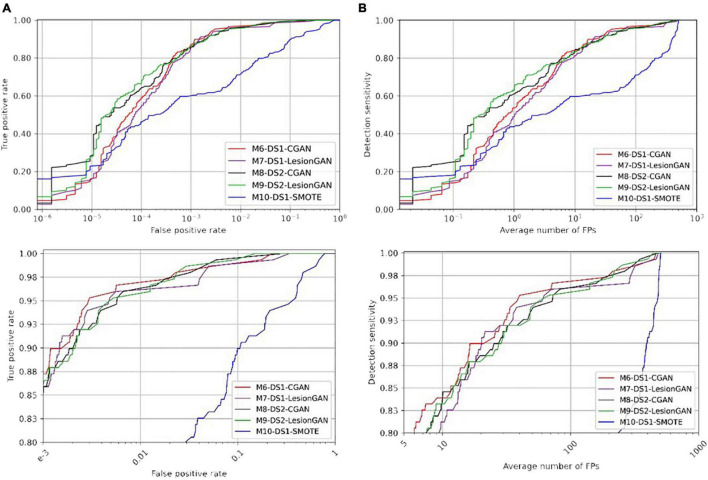
Receiver operating characteristic (ROC) **(A)** and free-response ROC **(B)** curves for cerebral microbleed classification on the whole unseen MRI image (DS2). The bottom panel shows the zoomed version.

To further compare performances, we applied the same trained models on the whole MRI image from DS2. Figure 9 shows the ROC and FROC curves, and Table 6 presents the result of CMB classification on the whole MRI image for DS2. CMB screening was done on DS2 using RST with a radius range and strictness degree adapted to take into account the different resolution. The standard deviation threshold of RST was also adapted to produce the same number of candidates as in DS1 (∼7,000 per scan).

**TABLE 6:** Results of cerebral microbleed (CMB) classification on the whole MRI image.

Training on the sCMB from DS1, test on the DS2				Training on the sCMB from DS2, test on the DS2			
**Training model**	**AUC**	**Sensitivity (95%)**		**Training model**	**AUC**	**Sensitivity (95%)**	
		**Spe**	**Number of FP/scan**			**Spe**	**Number of FP/scan**

M6-DS1-CGAN	0.9971	**0.9970**	**39**	M8-DS2-CGAN	0.9975	0.9941	72
M7-DS1-LesionGAN	0.9958	0.9950	61	M9-DS2-LesionGAN	**0.9980**	0.9950	62
M10-DS1-SMOTE	0.9560	0.6170	480				

*AUC, area under receiver operating characteristic curve; Spe, specificity. Bold represents the best score for each criteria.*

Regarding Table 6 (left and right panels), the best performance in terms of AUC was again achieved by M9-DS2-LesionGAN (0.9980) and M8-DS1-CGAN (0.9975), followed by M6-DS1-CGAN and M7-DS1-LesionGAN with 0.9971 and 0.9958, whereas the lowest AUC was achieved by M10-DS1-SMOTE (0.9560).

In terms of specificity and number of FP for 95% sensitivity, M6-DS1-CGAN was the best model, except when retraining the classifier using DS2, for which M9-DS2-LesionGAN was superior. M10-DS1-SMOTE obtained again the worst performance with 0.6170 specificity and 480 FP.

In summary, to achieve less than 10 false positive detections per scan, LesionGAN (M9-DS2-LesionGAN) had the best sensitivity (84%), while standard augmentation techniques (M10-DS1-SMOTE) could achieve no more than 60% (from Figure 9B bottom panel).

## Discussion

We proposed a novel 3D conditional GAN model to create synthetic microbleeds and to train a classifier (CNN) that achieved high performance on both patch classification and lesion detection from the whole SWI where it achieved less than 10 false positive detections per scan with a sensitivity of 90% (FROC curve in Figure 7B). We applied our trained GAN model on unseen MRI images and showed that it can generate high-quality fake lesions without using any ground truth (real lesion) for training. The proposed synthetic data generation model compared favorably to other data augmentation methods.

Some important features of our model are the use of GAN conditions. We included the volume as a condition to force the synthetic lesions to be created with a desired volume distribution. This is important when the training data comprises real lesions with a disease- or population-specific distribution of volume—for example, in DS2, the lesions were bigger than in DS1, and by sampling from the real DS2 distribution, we could train a classifier specifically for DS2. A larger dataset encompassing a broader range of demographics and diseases would allow one to determine a distribution of lesion volumes, presumably generalizing the performance of a lesion detector for a broader clinical use. The second condition that improved the performance in our experiments was to provide the background, where the lesions will be added, to the generator so that lesions could be adapted to the surrounding tissue. We hypothesized that lesions close to a vessel, for example, might have a slightly different shape or appearance compared to lesions close to sulci. It was challenging to study whether this was true, but our results (comparing the reported results in Tables 2, 3) show that the extra information provided by the background improved the classifier performance.

It is challenging to blend synthetic lesions to a healthy background without creating artifacts that would be easily learnt by a classifier. The early version created visible dark or bright patch artifacts when the synthetic lesion mask *G*(*z*) multiplied a brain location: the GAN would not converge to creating *G*(*z*) with unity on the outer edge, thereby creating a visible step in intensity. Adding a border loss solved this problem and, when multiplying by *G*(*z*), the synthetic lesions blended completely within the background.

Our proposed LesionGAN has advantages over similar synthetic lesion methods. By using a multiplicative lesion mask *G*(*z*), synthetic lesions could be added on any location of a healthy scan with different shape and volume without requiring a binary mask as proposed by others (Jin et al., 2018; Faryna et al., 2021). As a result, the synthetic lesions generated are independent of the accuracy of the real lesion segmentation used to produce the binary masks. Compared to Faryna et al. (2021), by creating a 3D lesion mask from sampling the latent space, there is no need for translating patches, thus saving processing time. Our method is also able to create multiple synthetic lesions for a given location.

In a previous work, we proposed to create synthetic lesion using an analytical model. A Gaussian blob was randomized to create a variety of shape and appearance (Momeni et al., 2021). The advantage of the analytical model is the non-reliance on ground truth segmentation, although in practice the hyperparameters of the methods (e.g., Gaussian shape parameters) were set up using observed real lesions. In contrast, our proposed LesionGAN is parameter-free and learns the shape and appearance of real lesion through the competition with the discriminator.

False positive might reveal missed lesions by an expert. Out of the 203 possible CMB from 75 scans marked by experts in DS1, the highest number was detected from M4-DS1-LesionGAN with 118 possible CMB, followed by M3-DS1-CGAN, M1-DS1-Analytical, and M2-DS1-GAN with 111, 110, and 108, respectively. In comparison, M5-DS1-SMOTE detected only 77 lesions marked as possible. This suggests that our proposed approach was able to generalize better than what linear combination of real lesion (SMOTE) could do. It also suggests that performance evaluation using cross-validation (train/test split) overestimates the performance of methods relying only on real lesions for training. Our experiments also demonstrate that reporting performance on patches does not translate to the performance needed to assess clinical use case. A detection on whole scan, reporting FP per scan, with a cross-validation across subjects should be preferred.

Our classifier performance compared favorably with published studies. Two existing methods had better results (Dou et al., 2016; Liu et al., 2019) but cannot be compared fairly. Dou et al. (2016) adopted a complex classifier prone to overfitting, especially when working with a small dataset (20 subjects). We could not find any report of performance evaluation when a method was trained and tested on a different cohort, like we have described in this manuscript. Liu et al. (2019) reported a very low number of FP (∼2), which is likely due to using multiple MRI sequences (QSM and SWI), including the phase information (four input channels). The extension of our method to multiple MRI sequences would likely yield better performance and will be part of future work. Moreover, it is difficult to compare performance between methods because of the different diseases considered: dementia, in our case, vs. stroke (Liu et al., 2019), hemodialysis, and traumatic brain injury (Dou et al., 2016). Ideally, each method should be tested on various diseases and MRI acquisitions. However, compared to a recent CycleGAN approach (Faryna et al., 2021), our synthetic data approach had better performance: 9.28 FP per patient compared to 27.4 and a higher specificity for the same sensitivity.

## Conclusion

An adversarial generative model of microbleed can create large training datasets with synthetic lesions and improve the performance and generalization of lesion detection using deep learning. We have proposed an approach that allows the creation of a training dataset for a new unseen cohort with no need for ground truth. We proposed a conditional GAN model to generate CMB masks. As inputs, to the generator, we included background information and target volume so that the synthetic lesions generated could be customized to location and volume. The mask of the synthetic CMB blends within the background at any new location allows one to create large datasets for the training classifier. Those synthetic lesions can then be added to any datasets without requiring any ground truth. By including more information to the generator, we hypothesize that synthetic lesions could be controlled for demographics, MRI sequences, and pathologies, allowing even greater generalization than what we studied in this manuscript, and is the focus of our future work.

## Data Availability Statement

The datasets presented in this study can be found online: https://doi.org/10.25919/aegy-ny12.

## Ethics Statement

The study was reviewed and approved by the Austin Health Human Research Ethics Committee and 174 St. Vincent’s Health Research Ethics Committee. Written informed consent was obtained from the participants.

## Author Contributions

SM: data analysis, programming, and manuscript writing. AF: data preprocessing. LL: data analysis. PY: clinical interpretation and visual rating providing the ground truth. CR: clinical interpretation and analysis, and experimental design. YG and AL: machine learning design and data analysis. OS: study design, machine learning design, data analysis, and manuscript writing. All authors contributed to the article and approved the submitted version.

## Conflict of Interest

The authors declare that the research was conducted in the absence of any commercial or financial relationships that could be construed as a potential conflict of interest.

## Publisher’s Note

All claims expressed in this article are solely those of the authors and do not necessarily represent those of their affiliated organizations, or those of the publisher, the editors and the reviewers. Any product that may be evaluated in this article, or claim that may be made by its manufacturer, is not guaranteed or endorsed by the publisher.

## Footnotes

1
https://valdo.grand-challenge.org/

2
http://aehrc.github.io/Mirorr/
